# Adventitial Fibroblasts in Aortic Aneurysm: Unraveling Pathogenic Contributions to Vascular Disease

**DOI:** 10.3390/diagnostics12040871

**Published:** 2022-03-31

**Authors:** Cameron D. A. Mackay, Anshul S. Jadli, Paul W. M. Fedak, Vaibhav B. Patel

**Affiliations:** 1 Department of Physiology and Pharmacology, Cumming School of Medicine, University of Calgary, Calgary, AB T2N 4N1, Canada; cameron.mackay1@ucalgary.ca (C.D.A.M.); anshul.jadli@ucalgary.ca (A.S.J.); 2 Libin Cardiovascular Institute, University of Calgary, 3330 Hospital Drive NW HMRB-G71, Calgary, AB T2N 4N1, Canada; paul.fedak@gmail.com; 3 Section of Cardiac Surgery, Department of Cardiac Sciences, Cumming School of Medicine, University of Calgary, Calgary, AB T2N 4N1, Canada

**Keywords:** aortic aneurysm, fibroblast, myofibroblast, aortic adventitia, adventitial remodeling, fibrosis

## Abstract

Aortic aneurysm (AA) is a degenerative vascular disease that involves aortic dilatation, and, if untreated, it can lead to rupture. Despite its significant impact on the healthcare system, its multifactorial nature and elusive pathophysiology contribute to limited therapeutic interventions that prevent the progression of AA. Thus, further research into the mechanisms underlying AA is paramount. Adventitial fibroblasts are one of the key constituents of the aortic wall, and they play an essential role in maintaining vessel structure and function. However, adventitial fibroblasts remain understudied when compared with endothelial cells and smooth muscle cells. Adventitial fibroblasts facilitate the production of extracellular matrix (ECM), providing structural integrity. However, during biomechanical stress and/or injury, adventitial fibroblasts can be activated into myofibroblasts, which move to the site of injury and secrete collagen and cytokines, thereby enhancing the inflammatory response. The overactivation or persistence of myofibroblasts has been shown to initiate pathological vascular remodeling. Therefore, understanding the underlying mechanisms involved in the activation of fibroblasts and in regulating myofibroblast activation may provide a potential therapeutic target to prevent or delay the progression of AA. This review discusses mechanistic insights into myofibroblast activation and associated vascular remodeling, thus illustrating the contribution of fibroblasts to the pathogenesis of AA.

## 1. Background

Aortic aneurysm (AA) is defined by an irreversible, pathological expansion of the aortic diameter accompanied by a thinning of the aortic wall [1,2,3,4]. Primarily affecting elderly individuals, this irreversible disease is usually asymptomatic until the aorta ruptures [2,4,5]. It is estimated that the total mortality of ruptured AA is 80%, while 32% of individuals die before reaching the hospital [6]. The incidence of AA is increasing among elderly individuals [7], and it has been estimated that over one million Americans are currently living with abdominal aortic aneurysm (AAA), the most common type of AA [7,8]. Smoking is the leading risk factor for AA; however, older age, high blood pressure, Caucasian race, coronary artery disease, and family history are some of the predisposing risk factors for the onset of AA [4,9,10]. Biological sex is another risk factor for AA since males exhibit higher rates of AAA (as high as 6:1 males to females) and, to a lesser extent, thoracic AA (TAA) (60:40), while females have worse outcomes in both AAA and TAA [8,10,11,12,13,14,15]. Females have increased rates of aneurysm development and rupture at lower diameters when compared to males, which persist despite controlling for body size [10,11,12,13,14,15,16]. Despite the substantial burden caused by this disease, there remain no pharmacological treatments to limit the progression of the disease or to prevent rupture [1,2,3,5]. The clinical management of AA using beta-blockers, calcium channel blockers, and angiotensin-converting enzyme (ACE) inhibitors limit AA progression but do not lead to reductions in aneurysm growth or the risk of rupture [5,17]. Clinically, AAs can be surgically treated if the risks of rupture or symptoms are predicted to be higher than the risks associated with surgery [4,5]. Surgical options include endovascular repair using stents or open surgical repair; however, these surgical operations carry a significant risk of perioperative mortality [2,4,5,9,18,19]. Postoperative complications occur in over one-third of patients, and they include acute kidney injury, wound infection, stroke, intestinal ischemia, atrial fibrillation, myocardial infarction, and seizures [2,4,5,9]. The lack of clinical interventions and unknown pathophysiology demonstrate the need for the development of pharmacological treatments to prevent or halt the progression of the disease and minimize the need for surgery [1,2,4,5]. Thus, more research into the molecular mechanisms underlying the progression of this deadly disease is paramount.

Since AA is often asymptomatic, many AAs are diagnosed incidentally through imaging unrelated to the aortic conditions [4,5,20,21,22]. Computed tomography (CT) and magnetic resonance imaging (MRI) are the most accurate options to assess the development and monitor the progression of AA [4,20,21]. Echocardiography can also be used as a cost-effective and more accessible diagnostic tool, thus making it an excellent option for screening [4,20,21,22]. However, it has been reported that measurements of aorta diameter can vary by 0.2–0.4 cm between echocardiography, CT, and MRI [4,20,22]. The benefits and limitations of these diagnostic tools have been extensively reviewed previously [4,21].

## 2. Current Understanding of Mechanisms Underlying AA

AA is characterized by progressive dilatation of the aorta, with adverse vascular remodeling resulting in weakening of the aortic wall and, subsequently, leading to dissection or rupture [1,2,5,7,23]. The wall of the aorta is divided into three layers. The innermost layer is the tunica intima, which is composed of endothelial cells and basement membrane. The middle layer, the tunica media, contains smooth muscle cells (SMCs) and layers of elastin fibers. The tunica adventitia, the outermost layer, primarily comprises fibroblasts [3]. The aorta is a dynamic organ that constantly responds to changes in its environment by turning mechanical stimuli, such as shear stress and chemical signals, into biological responses [4,24]. Cells in the aortic wall can respond to biomechanical stimuli through changes in protein synthesis, the rates of migration, proliferation, and cell differentiation [1,24,25,26,27]. While necessary for homeostasis, the overactivation of many cellular signaling pathways contributes to the progression of AA.

The renin–angiotensin system (RAS) is an essential regulator of blood pressure and the balancing of fluids and electrolytes. However, the overactivation of RAS and increased levels of angiotensin II (Ang II) have been shown to promote the progression of both AAA and TAA [1,2,24,25,26,27,28,29,30,31]. By interacting with the Ang II type 1 receptor (AT1R), Ang II can elicit a potent physiological response that increases blood pressure, which leads to changes in the expression of genes responsible for inflammation, ECM degradation, angiogenesis, proliferation, apoptosis, and cell cycling, resulting in the onset of AAA [26,27,28,29]. Transforming growth factor-beta (TGF-β) is a cytokine that plays a key role in modulating a variety of cellular processes, and it is often considered a potent activator of the inflammatory cascade [25,28,29,32,33,34]. The role of TGF-β as a modulator of “protective” or “pathogenic” pathways remains controversial, with evidence supporting either possibility [28,29]. This suggests that TGF-β signaling is integral to the maintenance of cardiovascular homeostasis [35]. TGF-β can signal through canonical or SMAD-dependent pathways, which are upregulated in mouse models of TAA and promote fibrosis during adventitial remodeling [26,29,36,37,38,39]. On the contrary, in AAA, TGF-β can activate numerous signaling pathways through its non-canonical or SMAD-independent pathways, which are thought to elicit a protective effect [40,41,42]. These pathways include phosphatidylinositol-3 kinase (PI3K) and mitogen-activated protein kinase (MAPK), among many other pathways [26,40,41,42]. In vascular SMCs, Ang II is also able to activate the SMAD pathway and the MAPK pathway independent of TGF-β [24,43]. Although various signaling pathways have been identified to be associated with the progression of AA, the cause-and-effect relationship between signaling pathways and the pathogenesis of AA remains unclear. A large amount of crosstalk between these pathways further hinders our understanding of AA pathogenesis. Thus, extensive research focusing on the underlying molecular mechanisms involved in the formation and progression of AA is highly warranted. A better understanding of the molecular pathophysiology of AA will enable us to identify novel targets with therapeutic potential to combat its progression.

**Figure 1:**
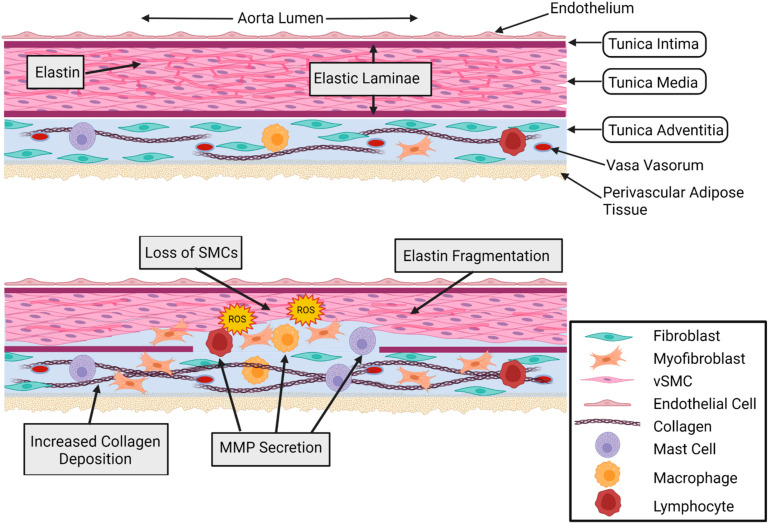
Healthy and aneurysmal aorta cross-sections. Comparison of healthy (**Top**) and aneurysmal (**Bottom**) aortic wall cross-sections. The healthy aortic wall shows an organized medial layer and no inflammatory cell infiltration. In the adventitial layer, there is minimal myofibroblast presence, low levels of inflammatory cells, and homeostatic levels of collagen. The aneurysmal aortic wall shows thinning of the medial layer, SMC loss, elastin fragmentation, inflammatory and mesenchymal cell infiltration of the medial layer, fibroblast conversion to myofibroblasts, reactive oxygen species (ROS), increased inflammatory cell presence, increased collagen deposition, and increased MMP secretion. Cell types are indicated in the figure legend. Figure created using Biorender.com accessed on 14 January 2022.

Various biological processes have been identified to contribute to the progression of AA, such as perivascular fibrosis, vascular wall remodeling, the loss of elastin, inflammation, oxidative stress, and the apoptosis of vascular SMCs [24,25,26]. Extracellular matrix (ECM) consists of elastin, collagen, glycoproteins, and proteoglycans, and it is essential for maintaining elasticity and structural integrity [44,45,46,47]. ECM degradation through the upregulation of matrix metalloproteinases (MMPs) and the downregulation of endogenous MMP inhibitors is observed in TAA, as well as in AAA [23,39,44,46,48]. Distinct MMPs are secreted by endothelial cells, SMCs, and fibroblasts in the healthy aorta, while inflammatory cells, such as macrophages, can produce additional MMPs [26]. The loss of ECM components leads to the compromised structural integrity of the aortic wall, which is exacerbated by increased SMC apoptosis [32,33,34]. The infiltration of inflammatory cell types is a critical component of AA progression [11,33,34,35,49,50]. Macrophages accumulate in the adventitial layer during AAA formation and secrete MMPs, cytokines, and chemokines [26,51,52,53,54]. T cells and B lymphocytes have also been implicated in contributing to the progression of AA [55,56,57]. High levels of reactive oxygen species (ROS) are observed in human TAA and AAA tissue samples [32,34,35,50,58]. ROS are thought to contribute to the progression of AA by activating MMPs and the transcription of proinflammatory genes while also promoting apoptosis [25,28,34,35,55]. Overall, these processes lead to maladaptive remodeling of the vessel wall and a loss of structural integrity, resulting in aortic dilatation and a progressive decline in vascular function (Figure 1).

Although a familial history of AAA is a risk factor for the development of AAA, suggesting a genetic component, specific genetic causes are not well understood [59]. Mutations in some loci have been identified as increasing the risk of AAA [59,60]. For example, various mutations in the 20q13.12 locus, which harbors the gene for MMP9, have been identified to increase the risk of AAA development [60]. Being a potent type IV collagenase and gelatinase, MMP9 degrades ECM; the upregulation of MMP9 in end-stage AAA corroborates its role in ECM remodeling in AAA progression [60,61]. However, there are other genes within this locus that encode other proteins, including PCIF1 and ZNF335; the roles of these proteins in AA remain to be studied [60]. Further research into the genetic factors underlying AAA is needed to identify the pathogenetic mechanisms and to identify individuals who are at risk.

However, unlike AAA, TAA has a strong genetic component in Marfan’s syndrome (MFS), Loeys–Dietz syndrome (LDS), and Ehlers–Danlos Syndrome (EDS), although these account for less than half of TAA incidences [9,14,59,62]. MFS is a multisystem syndrome involving a mutation in fibrillin-1, which is a crucial ECM component [59,63]. Fibrillin-1 regulates the availability and activity of TGF-β, and in MFS, dysfunctional fibrillin-1 leads to the overactivation of TGF-β, which plays a direct role in TAA pathogenesis [62,63,64,65]. LDS-associated mutations also alter TGF-β signaling [59]. LDS can manifest from mutations in various components of the TGF-β signaling cascade, including TGF-β receptor I (TGFBR1) or TGF-β receptor II (TGFBR2), TGF-β2, TGF-β3, SMAD2, and SMAD3 [14,59,62,65]. Although some of these mutations are expected to decrease the activity of TGF-β, increased TGF-β activity is documented in LDS and is expected to contribute to TAA pathogenesis [59,63,65,66]. EDS arises from mutations in genes encoding collagen or collagen-modifying mechanisms resulting in the absence of functional collagen, leading to dysfunctional ECM and, ultimately, TAA [59,62,63,67]. Although these genetic syndromes have led to an increased understanding of the genetic causes of TAA, further research into genetic factors underlying TAA is critical to decipher the underlying mechanisms involved in TAA pathogenesis.

## 3. Physiologic Role of Adventitial Fibroblasts

Fibroblasts are the most abundant cell type in the tunica adventitia [57,68,69,70,71,72]. This layer has been understudied and overlooked compared to the tunica intima and tunica media. In addition to fibroblasts, the adventitia also contains immune cells, nerves, lymphatic vessels, ECM, and a microvascular network known as the vasa vasorum [57,68,69,72,73]. The adventitia was initially thought to be a structural component of the blood vessel, but the importance of the adventitia in regulating the function of the vessel wall has been delineated in recent years [68,69,70,71,72]. The vascular adventitia is now known to sense and respond to various stimuli by communicating with cells in the adventitia and adjacent tissue or by becoming activated in response to injury, inflammation, hypoxia, and mechanical stress [57,68,69,70,71,72,74]. It has also been suggested that the vascular adventitia may play a role in regulating vascular tone through ECM and the production of nitric oxide [69,73,75,76,77,78]. Furthermore, dysfunctional fibroblasts have also been shown to cause arterial stiffness in pulmonary arterial hypertension, leading to impaired vessel compliance [69,73,79]. Moreover, sympathetic nerves in the adventitia can lead to SMC contraction through the release and diffusion of noradrenaline from the adventitia to the media [73,77]. Emerging evidence suggests the critical roles of adventitial cells in the regulation of vascular structural integrity, as well as in functional homeostasis.

Fibroblasts are responsible for the secretion of ECM, which is essential for maintaining the structure of the blood vessel wall [68,71,72,74]. The most notable ECM elements produced by fibroblasts are collagen types I and III [72,73]. This was initially thought to be the only role of adventitial fibroblasts; however, it is now known that fibroblasts are dynamic cells capable of sensing and responding to environmental stresses [72,73,74]. Adventitial fibroblasts play an essential role in wound healing by activating, migrating, and proliferating to the site of injury and, subsequently, depositing ECM molecules and collagen, which provides a scaffold for other cell types to adhere and grow [34,80,81,82]. The process of the activation of fibroblast, immune, and progenitor cells leads to a significant change in the behavior of these cells, with increased proliferation and migration, increased ECM deposition, and the increased expression of contractile proteins [69,72,82,83]. Cytokines, chemokines, and growth factors can also be secreted, which can further change the phenotype of the vessel wall [69,72,82]. Importantly, this essential process is also implicated in various cardiovascular diseases, including AA [1,34,68,69,70,71,72,74,82,83,84,85,86]. Complicating the in vitro study of fibroblasts is the fact that there are currently no fibroblast-specific markers for identification. Additionally, fibroblasts tend to transform when removed from the tunica adventitia, which is thought to be mostly in response to mechanical stress in in vitro cell culture conditions [68,71,87,88,89,90,91]. Thus, improved methods to identify and maintain fibroblasts in culture are crucial to increase the translational applicability of cellular studies involving adventitial fibroblasts.

## 4. Myofibroblasts and Vascular Remodeling

**Figure 2:**
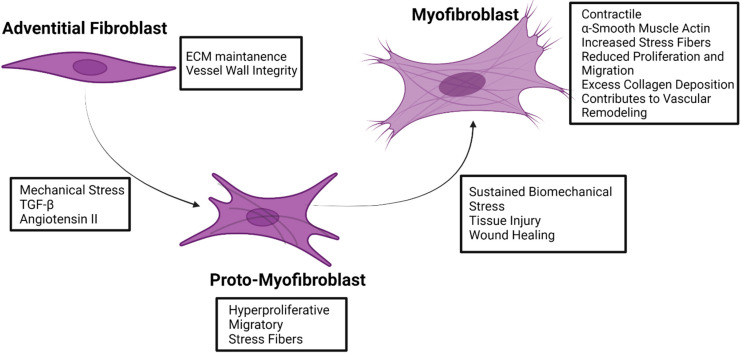
Fibroblast to myofibroblast transformation. Characteristics and inducers of transformation are indicated for adventitial fibroblasts, adventitial proto-myofibroblasts, and adventitial myofibroblasts. Figure created using Biorender.com accessed on 14 January 2022.

Central to the function of fibroblasts is the ability to respond to environmental stress. In response to various stresses, adventitial fibroblasts can transform into activated myofibroblasts, which are not typically found in healthy blood vessels [73,92]. During this transdifferentiation, fibroblasts undergo significant alterations to their behavior and transcriptional profile. This results in fibroblasts changing from a quiescent state into a mesenchymal state. Myofibroblasts exhibit increased contractile activity, migration and proliferation, cytokine and chemokine production, and ECM remodeling compared to fibroblasts [34,73,74,75,76,77,93]. Physiologically, this activation process of fibroblasts into myofibroblasts is necessary during wound healing and tissue repair [81,82]. As such, fibroblasts are thought to be the first cell type to become activated, reinforcing the idea of fibroblasts being a dynamic sensory cell type capable of causing physiological responses by secreting growth factors, chemokines, and cytokines and the activation of signaling pathways [72,73,74,76,77,85]. However, the transdifferentiation of fibroblasts into myofibroblasts is documented in various cardiovascular diseases, such as pulmonary arterial hypertension, heart failure, atherosclerosis, and AA [1,34,35,69,70,71,72,73,82,83,84,85,86,94,95,96,97,98]. A major identifier for fibroblast transformation into myofibroblasts is the expression of alpha-smooth muscle actin (α-SMA) [76,78,90,93]. In addition to α-SMA expression, myofibroblasts also develop stress fibers, which causes the myofibroblasts to exhibit contractile activity [82,93,99]. A precursor and intermediate phenotype during the fibroblast to myofibroblast transformation has been documented and termed the proto-myofibroblast [78,89,91,93,100]. These proto-myofibroblasts have stress fibers but lack α-SMA, and they have increased proliferative and migratory activities compared to activated myofibroblasts (Figure 2) [89,91,93,101].

Myofibroblasts are the primary cell type implicated in pathological perivascular fibrosis, which is characterized by maladaptive ECM remodeling and the accumulation of collagen in the adventitial region [34,35,82,102]. Collagen deposition is an adaptive physiological response to tissue damage and biomechanical stress. However, maladaptive, uncontrolled, and excessive collagen deposition leading to fibrosis is implicated in the pathophysiology of many inflammatory diseases of the cardiovascular system, including hypertension, pressure overload, myocardial infarction, cardiac inflammation, and heart failure [34,35,81,82,85,95,102]. In the heart, fibrosis is accompanied by the upregulation of pro-fibrotic factors, including TGF-β, and is characterized by a significant myofibroblast presence with maladaptive ECM remodeling [35,91,95]. Adventitial fibrosis is observed in response to chronic wall stress and hemodynamic changes in hypertension [73,103,104]. Being a vascular disease with hemodynamic changes at the forefront of its pathological basis, the activation of adventitial fibroblasts during AA development is a highly plausible proposition. Given the importance of the transformation of fibroblasts into myofibroblasts in the pathogenic process of various cardiovascular diseases, it is likely that, as research accumulates, the essential role of myofibroblasts in AA will be illuminated.

Although the precise role of myofibroblasts in AA is not currently understood, an increased myofibroblast presence is seen in AAA and TAA [1,100,105]. Since myofibroblasts have been identified as a major contributing factor to vascular remodeling in hypertension, myofibroblasts likely play a role in the maladaptive vessel remodeling seen in AA [30,90]. Adventitial remodeling is defined by a thickened adventitia, an increased number of fibroblasts, and the phenotypic switching of fibroblasts into myofibroblasts [30,70,77,90,96,97]. Increased collagen deposition by myofibroblasts leads to stiffening of the vessel wall [77,90]. Myofibroblasts also migrate and proliferate to the media and intima layers, where they can secrete chemokines, cytokines, and growth factors that potentiate the inflammatory response [30,73,77,90,96]. Progressive dilatation of the aortic lumen, the apoptosis of vascular SMCs, the loss of elastin, and ECM degradation weaken the aortic wall [30,31,73,106]. This increases vascular wall distensibility and causes the adventitia to become the primary structural support mechanism having to bear the mechanical load of the aortic wall rather than the elastic medial layer, which eventually leads to rupture [30,31,73,106]. Overall, this maladaptive remodeling of the vessel wall leads to a loss of contractile function and chronic inflammatory feedback mechanisms, although it is not known if this is a cause or an effect in the context of AA [30,31,70,73,74,76,90,96,97].

Aneurysm development in patients with Marfan syndrome (MFS) was found to exhibit the overexpression of TGF-β in MFS-cultured adventitial fibroblasts. This overexpression of TGF-β in MFS was associated with altered hyaluronan synthesis, impaired progenitor cell recruitment, and abnormal directional migration, thus resulting in impaired tissue repair and potential contribution to aneurysm development [107]. The activation of TGF-β signaling has been reported in patients with LDS-associated aortic aneurysms. Furthermore, the dexamethasone treatment of fibroblasts derived from patients with LDS demonstrated marked attenuation of aberrant elastic fiber production and collagen I secretion. These findings indicate the pathogenic implication of fibroblasts in aortic aneurysm development in patients with rare disorders [108]. Mitochondrial and metabolic abnormalities in adventitial fibroblasts have been indicated in other cardiovascular diseases, such as pulmonary arterial hypertension [98,109]. Moreover, vascular SMCs also exhibit metabolic abnormalities during hypertension [110] and intimal hyperplasia [111]. In the context of pulmonary arterial hypertension, mitochondrial fragmentation has been documented in many different cell types, including SMCs, endothelial cells, fibroblasts of the pulmonary artery, fibroblasts of the right ventricle, and cardiomyocytes [112,113,114]. Myofibroblasts produce a higher amount of ROS than fibroblasts [115]. Myofibroblasts are also able to increase the levels of superoxide radicals through increased NADPH oxidase activity [109,115]. Inhibiting mitochondrial fission in adventitial fibroblasts ameliorated transformation into myofibroblasts and mitochondrial fission-induced increases in ROS using a mouse model of pulmonary arterial hypertension [98]. TGF-β, Ang II, and shear stress also increase mitochondrial ROS [82,115]. As previously mentioned, high ROS levels are reported during AAA and TAA [26,40,41,42]. Thus, signaling pathways acting on the mitochondria and subsequent metabolic abnormalities may provide the link between fibroblasts, inflammation, oxidative stress, and AA.

## 5. Paracrine Effects of Adventitial Fibroblasts in the Pathogenesis of AA

Forming the outermost layer of the vessel wall, the adventitia is separated from media by the external elastic lamina and is believed to provide structural support to the vessel [79]. Fibroblasts represent the predominant cell population in the adventitia with small numbers of SMCs; adipocytes; pericytes; and resident immune cells, including lymphocytes, dendritic cells, and mast cells [116]. Human, in vitro, and in vivo experimental studies demonstrated the adventitia as a dynamic microenvironment in which adventitial fibroblasts and perivascular adipose tissue (PVAT) cells participate in the regulation of physiological and pathological processes [73,117]. Although adventitial fibroblasts are spatially associated with the outer layer of the aorta and are distant from the luminal surface, which is primarily associated with pathological initiation, fibroblasts exert profound functional influence on intimal and medial cell populations, i.e., ECs, macrophages, and SMCs [73]. A critical gap in knowledge is our understanding regarding whether alterations in adventitial fibroblasts reported in vascular diseases are a consequence of disease initiation and progression in the adventitia or whether adventitial dysfunction is an outcome of pathological changes associated with disease established in intima-media. This section highlights the adventitial fibroblast-mediated paracrine interactions with SMCs, ECs, and immune cells involved in the pathogenesis and progression of AA.

Using a mouse model with inducible fibroblast-specific deficiency of NADPH oxidase-2 (Nox2) (Fibro-Nox2KO mice), a recent study demonstrated the crucial role of fibroblast Nox2 in the development of Ang II-induced aortic vascular remodeling [118]. A fibroblast-specific deficiency of Nox2 displayed significantly attenuated vascular remodeling and hypertension in response to chronic Ang II infusion in mice. Mechanistically, the Ang II-induced vascular remodeling was attributed to the fibroblast Nox2-mediated regulation of paracrine signaling to medial SMCs via growth differentiation factor 6 (GDF6) [118]. In vitro experiments demonstrated Ang II-mediated adventitial remodeling via the phenotypic transformation of adventitial fibroblasts [119]. A co-culture of fibroblasts with ECs revealed the interaction between cell types and the inhibitory effects of ECs on the Ang II-induced phenotypic modulation of adventitial fibroblasts via the NO/cGMP signaling pathway [119].

**Figure 3:**
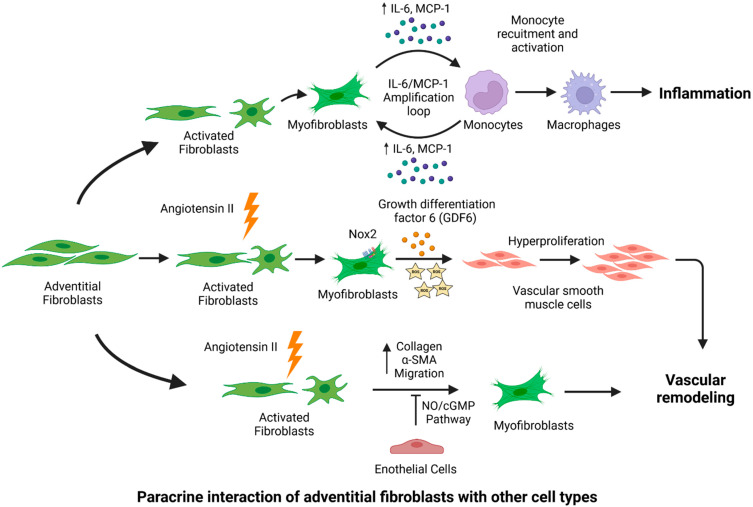
Fibroblast-mediated paracrine effects in the pathogenesis of aortic aneurysm (AA). Fibroblasts interact with other cell types in the aorta and may lead to the onset and progression of AA. Upon pathogenic stimuli or stress, activated adventitial fibroblasts interact with monocytes, which further amplify proinflammatory signaling via cytokine production. Inflammatory cytokines facilitate the recruitment and activation of monocytes into macrophages. Adventitial fibroblasts contribute to vascular remodeling via the generation of ROS and the GDF6-mediated hyperproliferation of VSMCs. Endothelial cells have inhibitory effects on fibroblasts, as they limit collagen production and myofibroblast migration. Figure created using Biorender.com accessed on 14 January 2022.

AA is generally characterized by profound adventitial remodeling preceded by an inflammatory response in the aortic wall [120]. Immune cell recruitment and accumulation in the adventitia may play an essential role in the pathogenesis of AAs, as published reports establish the adventitia as an efficient gateway for immune cell infiltration [121,122]. Activated aortic adventitial fibroblasts are reported to exacerbate vascular wall inflammation via the secretion of proinflammatory cytokines and chemokines [34,93,100]. Activated adventitial fibroblasts and macrophage interaction facilitate the IL-6/MCP-1 amplification loop, which accelerates vascular inflammation (Figure 3) [123]. Furthermore, in vitro and in vivo experiments demonstrated that interactions between fibroblasts and leukocytes in the aortic adventitia cause the aggravation of IL-6 secretion, thereby inducing local monocyte recruitment and activation into macrophages [124] and, consequently, promoting MCP-1 section, vascular inflammation, ECM remodeling, and aortic dissection in response to Ang II [124].

Despite recent scientific advances, our understanding of adventitial fibroblasts and their interaction with other aortic cells for the regulation of aortic homeostasis is limited. Further investigations are warranted to understand the precise role of adventitial aortic fibroblasts in aortic physiological processes and the pathological onset of AA.

## 6. Conclusions and Future Directions

Accumulating evidence suggests that extracellular vesicles (EVs) play a crucial role in the physiological and pathological processes of the cardiovascular system. By transferring effector molecules, such as nucleic acids, lipids, and proteins, to recipient cells, EVs facilitate intercellular communication. [125,126]. Currently, the diameter of EVs demarcates them into exosomes (50–100 nm), microvesicles (100–1000 nm), or apoptotic bodies (>1000 nm) [126]. Exosomes are of particular interest due to their nano-size and lack of immunogenicity, making these EVs less likely to trigger an immune response [126,127,128]. Much of the research on exosome-mediated communication in the cardiovascular system focuses on the heart, where cardiomyocytes, ECs, and fibroblasts, the major cell types of the heart, all produce and secrete exosomes [125,126]. Exosomal communication also occurs in the vasculature, and recent evidence suggests that exosome-mediated intercellular communication may play a role in AA. In a recent study, the inhibition of macrophage exosome production attenuated CaPO_4_-induced AAA development, suggesting that exosomes secreted by macrophages may play a role in the pathogenesis of AAA [129]. Another study showed that adventitial fibroblast-derived exosomes from hypertensive rats stimulated the migration of vascular SMCs through the exosome-mediated delivery of ACE [130]. Thus, exosomes may be the key to understanding intercellular communication between the cell types of the aorta, and they may underlie progressive, maladaptive vascular remodeling in AA. More research into exosomes and AA could lead to novel diagnostic techniques and treatment strategies.

Clinically, there is an unavailability of therapeutic alternatives to prevent the onset or delay the progression of AA [1,2,3,5]. Targeting fibroblasts could provide a key therapeutic option due to their pathogenic contribution to AA development. The maintenance of the quiescent phenotype of fibroblasts seen in healthy individuals or the reversal of activated myofibroblasts into fibroblasts has the potential to either limit or reduce the progression of the pathological remodeling of the aorta. Murine models of AA have shown that inhibiting adventitial remodeling and fibroblast activation abrogates AA development [98,131]. Comprehensive preclinical research targeting fibroblasts in AA is warranted. An extensive understanding of the role of fibroblasts in the pathogenesis of AA will provide novel therapeutic avenues.

Despite advances in AA research, the involvement of various cell types and associated signaling pathways hinders the precise understanding of the pathogenic mechanisms underlying the onset and progression of AA. The failure of the translation of the preclinical efficacy of pharmacological agents in clinical use indicates the need for further investigations to identify the pathogenic mediators of AA. Adventitial fibroblasts play an essential role in the structure and function of the vascular wall. Although detailed investigations delineating adventitial fibroblast-mediated complex interactions with other cell types of the aorta and signaling pathways triggering AA development are yet to be conducted, increasing evidence indicates the crucial role of adventitial fibroblasts in AA development. Physiologically, the transformation of fibroblasts into myofibroblasts is essential for wound healing, but the excessive activation and phenotypic switching of fibroblasts have been implicated in cardiovascular diseases, such as pulmonary arterial hypertension. More research is needed to pinpoint the direct and indirect mechanisms through which fibroblasts contribute to AA. Furthermore, technical challenges surrounding the research of adventitial fibroblasts are significant, which emphasizes the need for proper techniques for maintenance and markers for the identification of fibroblasts. The identification of adventitial fibroblast-mediated pathogenic alterations in signaling pathways and complex paracrine interactions with SMCs, ECs, and inflammatory cells may elucidate additional intricacies of the pathophysiology of AA onset and progression and, thus, help in the identification of novel therapeutic targets.
